# The mTOR inhibitor AZD8055 inhibits proliferation and glycolysis in cervical cancer cells

**DOI:** 10.3892/ol.2012.1058

**Published:** 2012-12-04

**Authors:** SHAORU LI, YAN LI, RUILI HU, WEIHUA LI, HAIFENG QIU, HONGHUA CAI, SHIJIN WANG

**Affiliations:** 1 Departments of Gynecology and Obstetrics, The First Affiliated Hospital of Xinxiang Medical University, Weihui, Henan 453100;; 2 Endocrinology, The First Affiliated Hospital of Xinxiang Medical University, Weihui, Henan 453100;; 3 Department of Gynecology and Obstetrics, Peking Union Medical College Hospital, Beijing 100730;; 4 Department of Obstetrics and Gynecology, International Peace Maternity and Child Health Hospital of the China Welfare Institute, School of Medicine, Shanghai Jiaotong University, Shanghai 200030, P.R. China

**Keywords:** cervical cancer, AZD8055, proliferation, glycolysis, mTOR signaling, anticancer activity

## Abstract

The aim of the present study was to determine the effect of AZD8055 on proliferation, apoptosis and glycolysis in the human cervical cancer cell line HeLa and to investigate the underlying mechanism(s) of action. HeLa human cervical cancer cells were treated with 10 nM AZD8055 for 24, 48 or 72 h. MTT was used to determine cell proliferation. Annexin V/propidium iodide staining was used to determine cell apoptosis analyzed by fluorescence-activated cell sorting (FACS). Glycolytic activity was determined by measuring the activity of the key enzyme lactate dehydrogenase (LDH) and lactate production. RNA and protein expression were examined by qRT-PCR and western blotting, respectively. Treatment with AZD8055 inhibited proliferation and glycolysis, and induced apoptosis in HeLa cells in a time-dependent manner. During the prolonged treatment with AZD8055, the phosphorylation of mammalian target of rapamycin (mTOR) C1 substrates p70S6K and phosphorylation of the mTORC2 substrate Akt were deregulated, suggesting that the activity of mTOR was downregulated. Furthermore, our study showed that the expression of miR-143 was upregulated in a time-dependent manner in HeLa cells treated with AZD8055. In summary, the present study reveals a novel antitumor mechanism of AZD8055 in HeLa human cervical cancer cells.

## Introduction

Cervical cancer is the third most common cancer in females. In 2008, 529,800 women were diagnosed with cervical cancer, accounting for 9% of all types of cancer diagnosed in females worldwide. Furthermore, cervical cancer is the fourth leading cause of mortality in females, accounting for 8% (275,100) of total cancer-related mortality rates in females worldwide in 2008 (1,2). Thus, developing an effective drug is of utmost importance.

Abnormal signaling pathways play crucial roles in the pathogenesis and progression of cancer (3). Mammalian target of rapamycin (mTOR) is a serine/threonine protein kinase, which forms two distinct multiprotein complexes, mTORC1 and mTORC2. mTORC1 phosphorylates downstream proteins p70S6K (S6K), which are involved in protein translation, while mTORC2 phosphorylates Akt, increasing its enzymatic activity by 5- to 10-fold (4,5). Furthermore, mTOR functions as a sensor of mitogen, energy and nutrient levels, and is a central controller of cell growth and a negative regulator of autophagy (6). AZD8055 is a first-in-class orally available, potent and specific inhibitor of mTOR kinase activity, and shows a promise for suppressing tumor growth (7). Currently, AZD8055 is in phase I clinical development. Understanding the molecular mechanisms of AZD8055 on human cervical cancer HeLa cells may facilitate the development of strategies for the therapy of cervical cancer.

Energy metabolism is considered to be a promising target for cancer therapy. In multiple types of malignant tumors, energy metabolism is aberrantly upregulated. Cancer cells preferentially metabolize glucose through glycolysis even under adequate levels of oxygen, which is known as the Warburg effect.

miRNAs are small endogenous and non-coding RNAs that inhibit gene expression via interaction with target sites in the 3’-untranslated region (UTRs) of mRNAs. miRNAs play important roles in regulating multiple biological processes, including the pathogenesis of a variety of human types of cancer (8). Lee *et al*(4) reported that miRNA deregulation may play a key role in the malignant transformation of cervical squamous cells. Accumulating evidence has shown that the expression of miR-143 is decreased in multiple types of cancer, and has been shown to be associated with lower survival rates and poor prognostic features (5,9).

The aim of the present study was to elucidate the role of AZD8055 in cervical cancer cells. We treated the cervical cancer cell line HeLa with AZD8055 for different durations to dynamically observe the effect of AZD8055 on cell proliferation, glycolysis and apoptosis. We also explored the effect of AZD8055 on the expression of miR-143 as well as several important enzymes involved in growth control and energy metabolism.

## Materials and methods

### Materials and reagents

The human cervical cancer cell line HeLa was purchased from the American Type Culture Collection (ATCC, Manassas, VA, USA). AZD8055 was obtained from Selleck (Houston, TX USA). Fetal bovine serum (FBS) was from Invitrogen (Carlsbad, CA, USA). Dulbecco’s modified Eagle’s medium (DMEM) and MTT were purchased from Sigma (St. Louis, MO, USA). The Annexin V-FITC Apoptosis Detection kit was purchased from Biovision (Mountain View, CA, USA). All antibodies were purchased from Santa Cruz Biotechnology, Inc. (Santa Cruz, CA, USA).

### Cell culture

The HeLa cells were cultured in DMEM containing 10% FBS, 100 U/ml penicillin and 100 *μ*g/ml streptomycin (Life Technologies, Carlsbad, CA, USA). The cells were cultured at 37°C in a humidified atmosphere with 5% CO_2_.

### MTT assays

Exponentially growing cells were seeded at 1×10^4^ cells/well in 96-well plates and incubated with AZD8055 at 10 nM for 24, 48 and 72 h, respectively. MTT (5 mg/ml) was added to each well and the plates were incubated for 4 h at 37°C. The formazan product was dissolved by adding 100 *μ*l DMSO to each well. The MTT absorbance value was detected at 490 nm with a micro-plate reader (Bio-Rad, Hercules, CA, USA).

### Flow cytometric analysis

For the detection of the percentage of apoptotic cells with Annexin V/propidium iodide (PI), following incubation with AZD8055 for 24-72 h, the HeLa cells were harvested and washed with cold PBS twice and then resuspended in 200 *μ*l binding buffer at a concentration of 1×10^6^ cells/ml. The cells were incubated with 10 *μ*l Annexin V-fluorescein isothio cyanate (FITC) and 5 *μ*l PI in the dark for 15 min. In total, 300 *μ*l binding buffer was then added in each tube prior to being analyzed with the flow cytometer.

### Measurement of glycolytic activity

The cellular glycolysis level was determined by measuring the cellular glucose uptake and lactate production. To measure glucose uptake, cells were washed with glucose-free medium and incubated in fresh glucose-free RPMI-1640 medium for 3 h, and then incubated with 0.2 Ci/ml ^3^H-2-deoxyglucose for 1 h. The glucose uptake represented by ^3^H radioactivity was determined by liquid scintillation counting and normalized by cell number. Lactate concentration in the culture medium was measured using the Lactate Acid Assay kit (Biovison). The protein expression of HK2, a key time-limiting enzyme in the activation of glucose, was also measured.

### Western blotting

Cells were lysed in cold RIPA lysis buffer. The protein concentrations were measured by a bicinchoninic acid (BCA) protein assay kit (Pierce Biotechnology, Rockford, IL, USA). The samples (20 *μ*g) were loaded and separated by SDS-PAGE and then electrophoretically transferred to PVDF membranes (Pall, New York, NY, USA). The membranes were probed with various antibodies according to the manufacturer’s instructions and visualized with peroxidase and an enhanced chemiluminescence system (Pierce Biotechnology).

### Statistical analysis

Data are expressed as the mean ± SEM. The statistical significance of the differences was determined by ANOVA followed by Student-Newman-Keuls (S-N-K) test or unpaired two-tailed t-tests. P<0.05 was considered to indicate statistically significant differences. Statistical analysis was performed using SPSS 17.0 statistical software.

## Results

### AZD8055 inhibits proliferation of human cervical cancer HeLa cells

**Figure 1.**
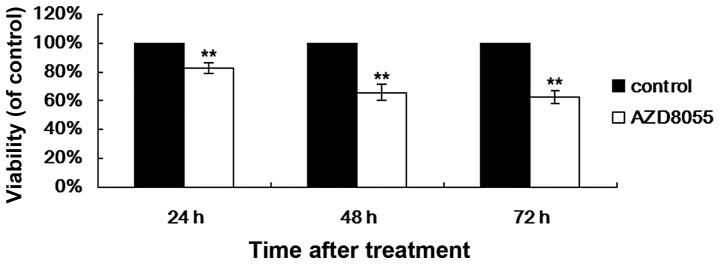
AZD8055 inhibits cell proliferation in human cervical cancer HeLa cells. An MTT assay was applied to examine the relative proliferation rate of HeLa cells after treatment with AZD8055 for 24, 48 and 72 h. During prolonged treatment with AZD8055, the relative cell proliferation rate gradually decreased. The difference between each group is statistically significant. ^**^P<0.01, compared with the control.

We first examined the effect of AZD8055 on the proliferation of HeLa cells. An MTT assay was applied to determine the relative proliferation rate of HeLa cells treated with AZD8055 for different durations (Fig. 1). Following AZD8055 treatment for 24, 48 and 72 h, the average cell proliferation rate was 82.97±3.74, 65.93±5.89 and 62.42±4.10%, respectively. These data demonstrate that AZD8055 time-dependently inhibited the proliferation of HeLa cells compared with controls (P<0.01 compared with the control).

### AZD8055 induces apoptosis of human cervical cancer HeLa cells

**Figure 2.**
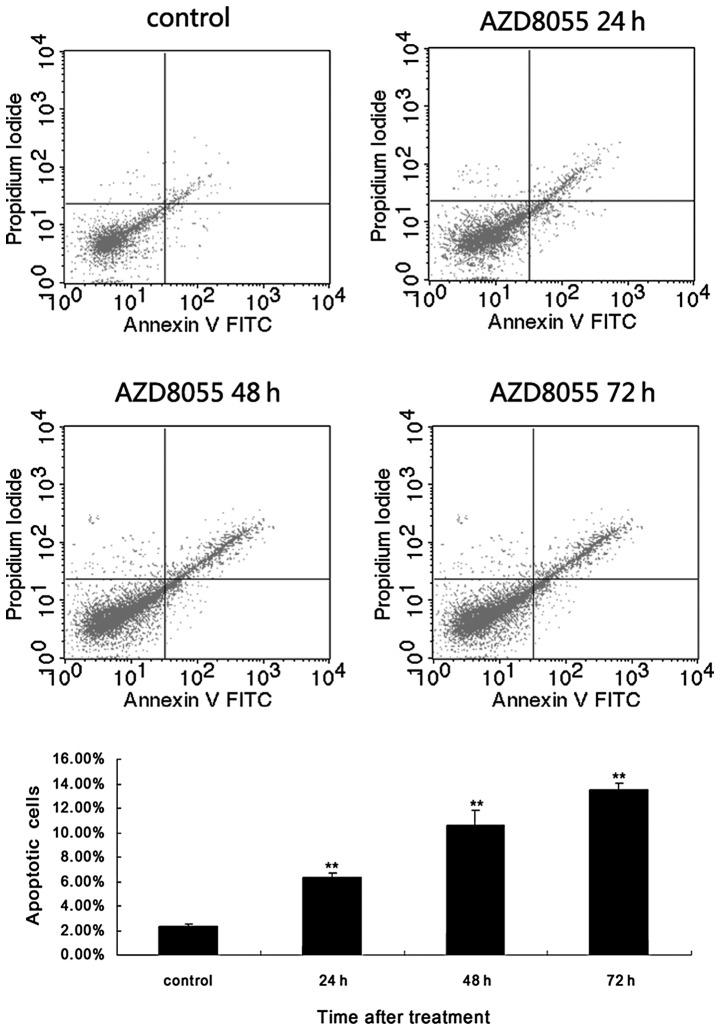
AZD8055 increases apoptosis in cervical cancer cells. Following treatment with 10 nM AZD8055 for 24, 48 and 72 h, HeLa cells were stained with FITC-conjugated Annexin V/PI. Percentage of Annexin V/PI-stained cells was determined by flow cytometry. Dot plot was divided into a quadrant: upper left, necrotic cells; upper right, late apoptotic cells; lower left, living cells; and lower right, early apoptotic cells. Graphs demonstrate the sum of cells in early and late apoptosis. Data are representative of 3 separate experiments. ^**^P<0.01, compared with the control. PI, propidium iodide.

The inhibition of cell proliferation by AZD8055 in HeLa cells may also be due to the induction of apoptosis. We therefore examined whether the antiproliferative effect of AZD8055 was accompanied by the induced apoptosis. HeLa cells treated with AZD8055 for different durations were analyzed using Annexin V/PI double-staining and flow cytometry analysis. As shown in Fig. 2, incubation with 10 nM AZD8055 increased the Annexin-positive cells from 2.31±0.17 to 6.37±0.37, 10.58±1.25 and 13.47±0.59% at 24, 48 and 72 h, respectively. Thus, AZD8055 time-dependently decreased the viability of HeLa cells. Moreover, AZD8055 significantly increased the early- and late-stage apoptotic fraction in cervical cancer cells, suggesting that suppression of cell proliferation by AZD8055 was partly due to the induction of apoptosis.

### AZD8055 inhibits glycolysis of human cervical cancer HeLa cells

**Figure 3.**
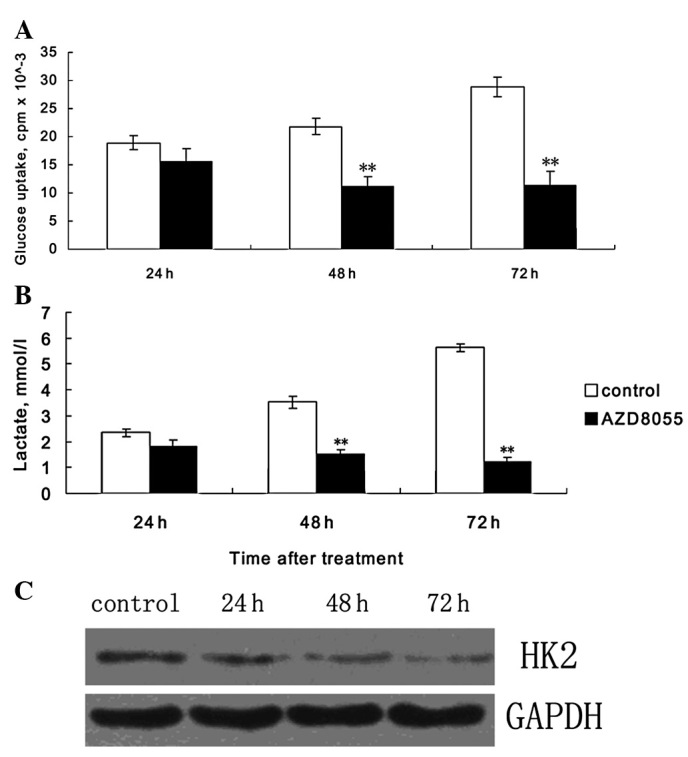
AZD8055 inhibits glycolysis of human cervical cancer HeLa cells. (A) The glucose uptake in HeLa cells was decreased significantly after the treatment with AZD8055 for 24, 48 and 72 h. (B) The lactate production in HeLa cells was downregulated significantly after the treatment with AZD8055 for 24, 48 and 72 h. (C) Following treatment with 10 nM AZD8055 for 24, 48 and 72 h, the protein level of HK2 in HeLa cells was examined using western blotting. ^**^P<0.01.

The level of glycolysis is always aberrantly upregulated in cancer, and is needed for the rapid proliferation of cancer cells. Thus, we further examined whether AZD8055 inhibited glycolysis in HeLa cells. We measured the cellular glucose uptake and lactate production in different groups. As shown in Fig. 3A and B, HeLa cells treated with AZD8055 for different durations showed decreased glucose uptake and lactate production, compared with controls. HK2 is a key time-limiting enzyme in glycolysis. Thus, we further examined the expression of HK2 by western blotting. As shown in Fig. 3C, during the treatment of HeLa cells with AZD8055, the expression of HK2 was time-dependently decreased, suggesting that AZD8055 may inhibit HeLa cell proliferation through inhibiting glycolysis at least partially by downregulating the expression of HK2.

### AZD8055 inhibits the activity of mTOR

**Figure 4.**
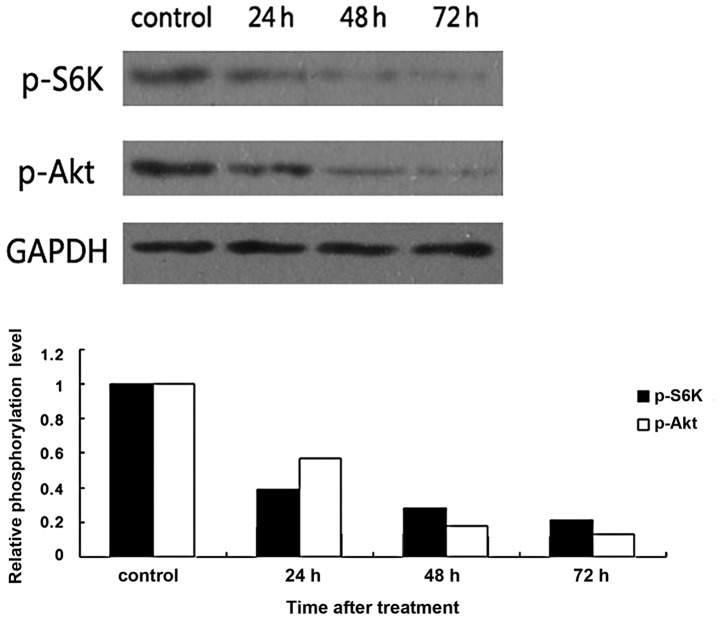
AZD8055 inhibits the activity of the mTOR signaling pathway. Western blot analysis of the phosphorylation of S6K and Akt in HeLa cells after treatment with 10 nM AZD8055 for 24, 48 and 72 h. GAPDH was used as a control. mTOR, mammalian target of rapamycin.

To investigate the molecular mechanism of AZD8055 on HeLa cells, we examined the effect of AZD8055 on the activity of mTOR, an important component of the PI3K/Akt/mTOR signaling pathway that plays a crucial role in the pathogenesis of cervical cancer (10). Our data showed that the phosphorylation of S6K and Akt were significantly reduced by AZD8055 in a time-dependent manner in HeLa cells (Fig. 4), which are the best characterized targets of the mTOR complex cascade. These findings suggest that AZD8055 may inhibit cervical cancer cell proliferation by repressing the mTOR pathway.

### AZD8055 upregulates the expression of miR-143

**Figure 5.**
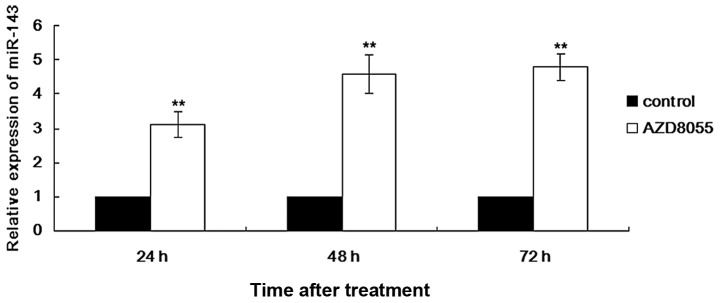
Relative expression of miR-143 in HeLa cells treated with AZD8055. After treatment with 10 nM AZD8055 for 24, 48 and 72 h, the relative expression of miR-143 in HeLa cells was determined. The histogram plots show the relative expression of miR-143 in different groups. ^**^P<0.01, compared with the control.

miRNAs play critical roles in cell biology, including regulating cell proliferation and apoptosis (11). In recent years, an increasing number of studies has reported that miRNAs are involved in the progression of cancer (12). mTOR was recently demonstrated to regulate the expression of miR-143 in lung adenocarcinoma cell lines (13). However, whether this regulating mechanism exists in cervical cancer remains unclear. Based on these findings, we further determined the expression of miR-143 in HeLa cells treated with AZD8055 for 24-72 h by qRT-PCR. As shown in Fig. 5, the upregulation of miR-143 by AZD8055 is time-dependent with the maximal increase (3.5-fold) achieved at 24 h of treatment. These results reveal a novel molecular mechanism of AZD8055/mTOR-mediated growth inhibition of cervical cancer cells.

## Discussion

The present study described AZD8055 as a potent mTOR inhibitor involved in multiple cellular responses, including inhibiting proliferation and inducing apoptosis in human cervical cancer HeLa cells. Notably, this is the first study to demonstrate that AZD8055 is able to reduce glycolysis levels in cancer cells. Our study suggested that AZD8055-mediated cervical cancer cell proliferation inhibition may occur through deregulating mTOR activity and upregulating the expression of miR-143, whose targets include HK2, a key time-limiting enzyme in the process of glucose activation.

mTOR is a protein kinase involved in the PI3K/Akt signaling pathway with a central role in the control of cell growth, proliferation, metabolism, survival and angiogenesis (14,15). It has been well established that the PI3K/Akt/mTOR signaling pathway plays a crucial role in cancer development. Multiple studies have reported that this signaling pathway is aberrantly activated in multiple types of cancer (16,17). As a result, the frequent dysregulation of this signaling pathway in cancer make it an important target in the treatment of multiple types of malignant tumors (18,19). However, only limited published data are available on new mTOR inhibitors and the detailed mechanism of AZD8055 in treating cervical cancer has yet to be fully understood. The present study showed that AZD8055 inhibits the phosphorylation of the mTORC1 substrates p70S6K and phosphorylation of the mTORC2 substrate Akt, suggesting that the activity of mTOR signaling was deregulated. These results indicated that the deregulation of the mTOR signaling pathway may be involved in the AZD8055-induced antiproliferative effects and apoptosis in cervical cancer HeLa cells.

The Warburg effect is a type of aberrant glucose metabolism common in cancer, which makes cancer cells less dependent on oxygen and promotes their survival under hypoxic conditions (3). Thus, glycolysis in cancer may become a promising target for the treatment of malignant tumors. In this study, AZD8055 time-dependently inhibited glycolysis in HeLa cells, indicated by the decreased LDH activity and lactate production. Additionally, the high glycolysis level in cancer is often associated with the aberrant overexpression of some key enzymes in glucose metabolism. Mathupala *et al*(20) reported that HK2 is involved in the maintenance of the malignant state of cancer. In fact, HK2 was able to promote the first step of glycolysis, thus, its overexpression would provide cancer cells with adequate glycolytic flux and further the shift towards aerobic glycolysis (21). Our data demonstrated that AZD8055 significantly deregulated the protein expression of HK2, providing a potential mechanism by which AZD8055 inhibited glycolysis in HeLa cells.

miRNAs are small endogenous non-coding RNAs, involved in the post-transcriptional regulation of gene expression by binding to the 3’-UTR of target mRNAs, which eventually leads to translational repression or mRNA degradation (22). miR-143 has been reported to play a crucial role in cancer development (23–26). The downregulation or loss of miR-143 may contribute to tumorigenesis, while forced expression of miR-143 may effectively inhibit the proliferation of cancer cells. Recently, Fang *et al* found that mTOR was able to inhibit the expression of miR-143, which further regulates cancer glycolysis via targeting HK2 in lung adenocarcinoma cell lines (12). In fact, HK2 has recently been demonstrated to be a direct target of miR-143 (27,28). In this study, the molecular experiments showed that AZD8055 downregulated the activity of mTOR, upregulated the expression of miR-143 and inhibited the expression of HK2 in HeLa cells. Based on these data, we suggested that AZD8055 inhibited the proliferation and glycolysis of HeLa cells partially through upregulating the expression of miR-143, which further deregulated the expression of HK2.

In conclusion, the present study provides a novel antitumor mechanism of AZD8055 in human cervical cancer cells. Our findings suggest that miR-143 may be an important potential target for cervical cancer treatment and AZD8055 may be a useful therapeutic agent for the treatment of human cervical cancer.
